# Phase Error Correction for Approximated Observation-Based Compressed Sensing Radar Imaging

**DOI:** 10.3390/s17030613

**Published:** 2017-03-17

**Authors:** Bo Li, Falin Liu, Chongbin Zhou, Yuanhao Lv, Jingqiu Hu

**Affiliations:** 1 Department of Electronic Engineering and Information Science, University of Science and Technology of China, Hefei 230027, China; libo702@mail.ustc.edu.cn (B.L.); zhouzcb@mail.ustc.edu.cn (C.Z.); yuanhaolv0203@163.com (Y.L.); hujq@mail.ustc.edu.cn (J.H.); 2 Key Laboratory of Electromagnetic Space Information, Chinese Academy of Sciences, Hefei 230027, China

**Keywords:** phase error correction, compressed sensing, approximated observation, radar imaging

## Abstract

Defocus of the reconstructed image of synthetic aperture radar (SAR) occurs in the presence of the phase error. In this work, a phase error correction method is proposed for compressed sensing (CS) radar imaging based on approximated observation. The proposed method has better image focusing ability with much less memory cost, compared to the conventional approaches, due to the inherent low memory requirement of the approximated observation operator. The one-dimensional (1D) phase error correction for approximated observation-based CS-SAR imaging is first carried out and it can be conveniently applied to the cases of random-frequency waveform and linear frequency modulated (LFM) waveform without any a priori knowledge. The approximated observation operators are obtained by calculating the inverse of Omega-K and chirp scaling algorithms for random-frequency and LFM waveforms, respectively. Furthermore, the 1D phase error model is modified by incorporating a priori knowledge and then a weighted 1D phase error model is proposed, which is capable of correcting two-dimensional (2D) phase error in some cases, where the estimation can be simplified to a 1D problem. Simulation and experimental results validate the effectiveness of the proposed method in the presence of 1D phase error or weighted 1D phase error.

## 1. Introduction

The synthetic aperture radar (SAR) is able to achieve image reconstruction with high resolution in both range and azimuth direction. However, higher range resolution of SAR requires wider bandwidth of signal, and thereby a higher sampling rate. Similarly, higher azimuth resolution requires longer synthetic aperture and higher sampling rate in azimuth direction. When both higher range and azimuth resolutions are expected, much higher sampling rate and larger volume of sampled data are required. It is thus a great challenge to design a high-resolution radar system hardware with limited cost.

Based on the theory of compressed sensing (CS) [1,2], both the sampling rate and the volume of sampled data can be reduced, if the reconstructed scene is sparse or compressible. The assumption made in the image reconstruction algorithm is that the observation matrix is known exactly. The observation matrix, however, depends on the mathematical model of the observation process. The uncertainties of the observation position will lead to the mismatch of the mathematical model and the observation matrix, which will cause phase error of the raw data, and thus the defocus of the reconstructed image.

The autofocus techniques, which are developed for correcting the phase error, are still attracting the interests of scientists. Chen et al. [3] proposed a method of SAR motion compensation based on parametric sparse representation to achieve autofocus of high-resolution SAR image, which assumed that the motion parameters of the radar were constant in each subaperture. Then the trajectory error can be corrected by the estimated motion parameters. Mao et al*.* [4] proposed a knowledge-aided two-dimensional (2D) autofocus approach, but the method did not utilize the CS theory or sparse regularization method to enhance the feature of the reconstructed image.

Many methods were proposed to deal with the model error in the case of CS-based radar imaging. Önhon et al. [5] proposed a sparsity-driven method for joint SAR imaging and phase error correction, which can remove either one-dimensional (1D) or 2D phase error. Kelly et al. [6] proposed a stable algorithm, which corrects phase error and reconstructs SAR image with compressively sampled data. An approach was proposed in [7], which can solve a joint optimization problem to achieve model error parameter estimation and SAR image formation simultaneously. Yang et al. [8] reported a method that can estimate the observation position error accurately and thus the quality of reconstructed image is improved significantly.

Up to now, the existing methods correcting the phase error of CS-based radar imaging were achieved by solving a two-step optimization problem, i.e., image formation processing and phase error estimation. The frameworks of CS-based image formation processing in [5,7,8] were formulated using exact observation functions, which were inefficient to be applied to the high-dimensional cases. The method proposed in [6] made a small aperture angle approximation, which hampered the application of high-resolution radar.

In [9,10], the approximated observation operators were proposed, which reduce the computational complexity and memory cost dramatically, and thus can be applied in high-dimensional cases efficiently. The approximated observation-based method proposed in [9] was called “range-azimuth decoupled method” in [10].

The intent of this paper is twofold. First, we propose a method to correct 1D phase error for approximated observation-based CS-SAR imaging by replacing the exact observation with the mentioned approximated observation in the two-step optimization framework. Then, a modified 1D phase error model is proposed to make the phase error model more precise, while the number of the unknowns does not increase in the phase error model.

The method proposed in this paper is capable of correcting the phase error in the case of approximated observation-based CS-SAR imaging. Compared with the existing methods mentioned above, the proposed method reduces the memory cost. The proposed method is achieved by solving a two-step optimization problem. In the step of image formation processing, the approximated observations can be derived from the inverse of Omega-K [11] and chirp scaling [12] algorithms for random-frequency waveform and linear frequency modulated (LFM) waveform, respectively. The images can thus be reconstructed by conducting the iterative thresholding algorithm (ITA) [9]. In the step of phase error estimation, the 1D phase error model, which can be conveniently applied to the cases of random-frequency and LFM waveforms without any a priori knowledge, is utilized first, and a weighted 1D phase error model is then proposed, which is able to model the phase error more precisely. When incorporating the a priori knowledge of the phase error structures, the method using the proposed weighted 1D phase error model can compensate the 2D phase error by solving a 1D problem. Accordingly, the weighted 1D phase error model achieves a better performance compared to the 1D phase error model, while the number of unknowns keeps the same. Furthermore, data redundancy will not be decreased for the weighted 1D phase error estimation compared with 1D phase error estimation.

This paper is organized as follows. Section 2 describes the signal models of radar imaging, along with model expressions for random-frequency and LFM waveforms. The inherent relationships between the geometric models and the phase error models are also introduced. In Section 3, a phase error correction method is proposed for approximated observation-based CS-SAR imaging, including the target reconstruction method, the phase error estimation approach, the memory cost analysis, the convergence analysis, and the computational complexity analysis. The simulation and experimental results are presented in Section 4 to validate the effectiveness of the proposed method. We conclude the work in Section 5.

Notation: We will use the subsequent notations in this paper. Vectors, matrices and operators will be denoted by bold lower case, bold upper case and roman upper case, respectively, e.g., f is a vector, S is a matrix, and M is an operator. Superscripts $S^{T}$, $S^{*}$ and $S^{H}$ denote the transpose, conjugate and Hermitian transpose of S, respectively.

## 2. Radar Imaging Model

### 2.1. Signal Model

In applications of strip-map SAR, wide bandwidth signals are always used to achieve the high range resolution. In this paper, we consider two types of signal waveforms, namely the random-frequency waveform [13] and the LFM waveform [8,12].

#### 2.1.1. Random-Frequency Waveform

To overcome the limitations of the stepped-frequency SAR system, a random-frequency SAR imaging scheme was proposed in [13]. In the strip-map SAR using stepped-frequency waveform [11], the demodulated baseband echo signal for a point target can be expressed as

(1) $$s\left(n\right)=g\cdot \exp\left[-j4\pi f_{c}\left(n\right)R/c\right]$$

where n=1,2,⋯,N is the n-th frequency point in one sequence, g is reflectivity coefficient of the point target, $f_{c}\left(n\right)$ is the frequency value of the n-th frequency, R is the range from radar to the point target, and c is the velocity of light. The stepped-frequency points are denoted as

(2) $$\begin{array}{cc} f_{c}\left(n\right)=f_{c}+n\Delta f, & n=1,2,\cdots ,N \end{array}$$

where Δf is the frequency interval and $f_{c}$ is the starting frequency.

The received signal of a radar is the superposition of echoes from all scatterers in the scene. Based on Equation (1), the received signal of the n-th pulse in the m-th sequence is given by

(3) $$s\left(m,n\right)=\iint_{G_{0}}g\left(x,y\right)\exp\left[-j\frac{4\pi f_{c}\left(n\right)R\left(m,x,y\right)}{c}\right]dxdy$$

where m=1,2,⋯,M denotes the m-th observation position, (x,y) is the coordinate of a target, g(x,y) is reflectivity coefficient of the target on (x,y), R(m,x,y) is the range from the radar to the target in the m-th observation position, and $G_{0}$ is the area illuminated by the wave beam. Here, referring to [8], we also assume that the transmitting period for each frequency is relatively short. Thus, the range from the radar to the scene is not changed in one observation.

There is a trade-off between resolution and imaging width [11]. If the scene is sparse or compressible, it is found [13] that the frequency points can be reduced and the imaging width can be increased, while the range and azimuth resolutions remain the same based on the theory of CS. The difference between stepped-frequency waveform and random-frequency waveform is the frequency interval. In the strip-map SAR using random-frequency waveform, $f_{c}\left(n\right)$ will be substituted by $f_{c}^{\prime }\left(n_{s}\right)$, $n_{s}=1,2,\cdots ,N_{s}$, and the sequence $f_{c}^{\prime }\left(n_{s}\right)$ is selected from the sequence $f_{c}\left(n\right)$ randomly. $N_{s}$ is generally much smaller than N. The relationship between the stepped-frequency points and the random-frequency points is expressed in matrix form as:

(4) $$f_{c}^{\prime }=f_{c}\Theta_{r}$$

where $f_{c}^{\prime }\in {\mathbb{C}}^{1\times N_{s}}$ and $f_{c}\in {\mathbb{C}}^{1\times N}$ denote the set of random-frequency and stepped-frequency points, respectively, and $\Theta_{r}\in {\mathbb{C}}^{N\times N_{s}}$ is the sampling matrix.

Substituting $f_{c}\left(n\right)$ by $f_{c}^{\prime }\left(n_{s}\right)$ in Equation (3), the received signal of the random-frequency SAR is expressed as:

(5) $$s\left(m,n_{s}\right)=\iint_{G_{0}}g\left(x,y\right)\exp\left[-j\frac{4\pi f_{c}^{\prime }\left(n_{s}\right)R\left(m,x,y\right)}{c}\right]dxdy.$$

We consider that the scene consists of a series of point scatterers. Based on Equation (5), the received signal of the random-frequency SAR will be rewritten as:

(6) $$s\left(m,n_{s}\right)=\sum_{k=1}^{K}g\left(k\right)\exp\left[-j\frac{4\pi f_{c}^{\prime }\left(n_{s}\right)R\left(m,k\right)}{c}\right]$$

where K is the number of point scatterers, g(k) is reflectivity coefficient of the k-th point, and R is the range from the radar to the k-th point in the m-th observation position.

#### 2.1.2. LFM Waveform

The LFM waveform is another kind of signal waveform, which can achieve wide bandwidth. Transmitted LFM waveform can be expressed as:

(7) $$s_{T}\left(t\right)=p\left(t\right)\exp\left(j2\pi f_{c}t\right)$$

where t is fast time, $f_{c}$ is the carrier frequency, and p(t) is denoted as:

(8) $$p\left(t\right)=\mathrm{rect}\left(\frac{t}{T}\right)\exp\left(j\pi \gamma t^{2}\right)$$

where rect(⋅) is the time window, and γ is the chirp rate.

In the strip-map SAR using LFM waveform, the demodulated baseband echo signal for a point target is given by:

(9) $$s\left(t\right)=g\cdot p\left(t-2R/c\right)\exp\left(-j4\pi f_{c}R/c\right)$$

where g is reflectivity coefficient of the point target, R is the range from the radar to the point target, and c is the velocity of light.

The received data are the superposition of echoes from all scatterers in the scene. Based on Equation (9), the demodulated echo data in the m-th observation will be expressed as:

(10) $$s\left(m,t\right)=\iint_{G_{0}}g\left(x,y\right)p\left[t-2R\left(m,x,y\right)/c\right]\exp\left[-j\frac{4\pi f_{c}R\left(m,x,y\right)}{c}\right]dxdy.$$

where g(x,y) is reflectivity coefficient of the target, R(m,x,y) is the range from radar to the target, and $G_{0}$ is the area of imaging. We consider that the scene is discretized, and then the received signal will be rewritten as:

(11) $$s\left(m,t\right)=\sum_{k=1}^{K}g\left(k\right)p\left[t-2R\left(m,k\right)/c\right]\exp\left[-j\frac{4\pi f_{c}R\left(m,k\right)}{c}\right]$$

where K is the number of scatterers. In practice, the continuous signal should be discretized. Then, the echo signal using LFM waveform is given by

(12) $$s\left(m,n\right)=\sum_{k=1}^{K}g\left(k\right)p\left[t\left(n\right)-2R\left(m,k\right)/c\right]\exp\left[-j\frac{4\pi f_{c}R\left(m,k\right)}{c}\right].$$

### 2.2. Phase Error Model

**Figure 1:**
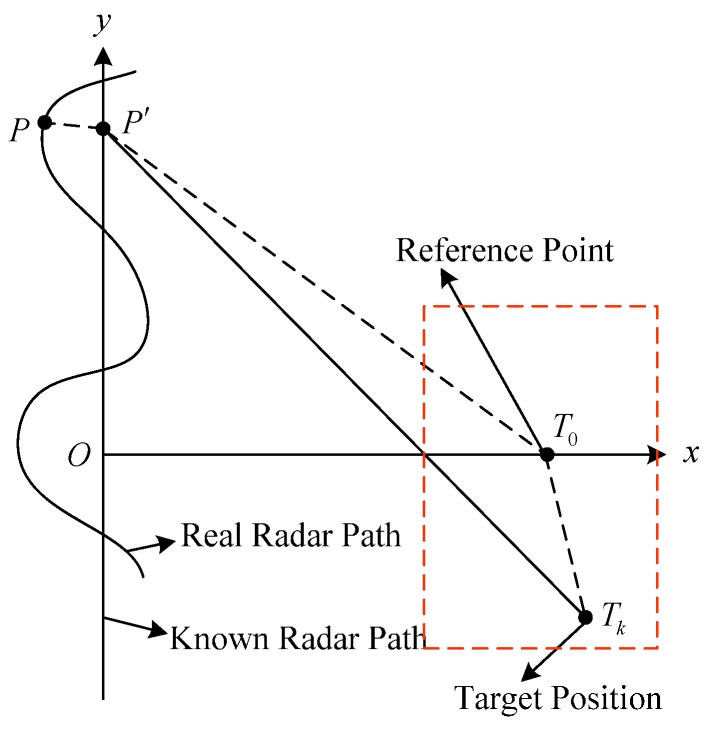
SAR imaging model.

After reviewing the existing signal models, the phase error models are analyzed as follows. In the SAR system, a radar transmits a series of pulse signals, and receives a series of echo data scattered from the illuminated scene. Considering 2-D slant-range plane [3], geometry of a SAR system is shown in Figure 1. The curved line indicates the real radar path, and the vertical axis indicates the known radar path, also nominated as “nominal path” in [3]. P is the real radar position, and $P^{\prime }$ is the known radar position. $T_{0}$ is the reference point of the scene, and $T_{k}$ is the k-th target in the illuminated scene.

Under the far-field assumption [14,15], range from the known radar position to the k-th target can be approximated as:

(13) $$\left|P^{\prime }T_{k}\right|\approx \left|P^{\prime }T_{0}\right|+\vec{T_{0}T_{k}}\frac{\vec{P^{\prime }T_{0}}}{\left|P^{\prime }T_{0}\right|}$$

where $\vec{P^{\prime }T_{0}}$ and $\left|P^{\prime }T_{0}\right|$ denote the direction vector and the range from $P^{\prime }$ to $T_{0}$, respectively.

Furthermore, radar platform position uncertainties will result in the difference between the known radar positon and the real radar position. Therefore, applying the far-field approximation, the range from the real radar position to the k-th target will be given by:

(14) $$\left|PT_{k}\right|\approx \left|P^{\prime }T_{0}\right|+\vec{T_{0}T_{k}}\frac{\vec{P^{\prime }T_{0}}}{\left|P^{\prime }T_{0}\right|}+\vec{P^{\prime }P}\frac{\vec{T_{0}P^{\prime }}}{\left|T_{0}P^{\prime }\right|}.$$

The third item on the right side in Equation (14) arises from the radar position uncertainties, which will result in phase error of echo data. Note that the third item is a common parameter for all the scatterers.

Considering the random-frequency waveform, the received signal of the random-frequency SAR can be expressed as Equation (6). Due to the radar position uncertainties, the received signal with model error must be modified as:

(15) $$s_{\varepsilon }\left(m,n_{s}\right)=\sum_{k=1}^{K}g\left(k\right)\exp\left[-j\frac{4\pi f_{c}^{\prime }\left(n_{s}\right)\left[R\left(m,k\right)+\Delta R\left(m,k\right)\right]}{c}\right]$$

where ΔR(m,k) arises from the radar position uncertainties. Referring to Equation (14), Equation (15) is approximated as:

(16) $$\begin{array}{ll} s_{\varepsilon }\left(m,n_{s}\right) & \approx \sum_{k=1}^{K}g\left(k\right)\exp\left[-j\frac{4\pi f_{c}^{\prime }\left(n_{s}\right)\left[R\left(m,k\right)+\Delta R\left(m\right)\right]}{c}\right]\\ & =\exp\left[-j\frac{4\pi f_{c}^{\prime }\left(n_{s}\right)\Delta R\left(m\right)}{c}\right]\sum_{k=1}^{K}g\left(k\right)\exp\left[-j\frac{4\pi f_{c}^{\prime }\left(n_{s}\right)R\left(m,k\right)}{c}\right]\\ & =\exp\left[-j\frac{4\pi f_{c}^{\prime }\left(n_{s}\right)\Delta R\left(m\right)}{c}\right]s\left(m,n_{s}\right) \end{array}$$

where $\exp\left[-j4\pi f_{c}^{\prime }\left(n_{s}\right)\Delta R\left(m\right)/c\right]$ arises from the radar position uncertainties, which will result in phase error in echo data and must be corrected in the phase error correction process. During the compensation of phase error, ΔR(m) must be estimated. Equation (16) is expressed in matrix form as:

(17) $$S_{\varepsilon }=D_{W1D}⊙S$$

where $D_{W1D}\in {\mathbb{C}}^{M\times N_{s}}$ denotes the phase error, ⊙ denotes the Hadamard product, and $S_{\varepsilon }\in {\mathbb{C}}^{M\times N_{s}}$ and $S\in {\mathbb{C}}^{M\times N_{s}}$ denote the echo data with phase error and without phase error, respectively. The phase error arising from radar position uncertainties is

(18) $$\begin{array}{ll} D_{W1D} & =\exp\left\{{\left[j\varphi_{W1D}\left(1\right),j\varphi_{W1D}\left(2\right),\ldots ,j\varphi_{W1D}\left(M\right)\right]}^{T}\frac{\left[f_{c}^{\prime }\left(1\right),f_{c}^{\prime }\left(2\right),\ldots ,f_{c}^{\prime }\left(N_{s}\right)\right]}{f_{c}}\right\}\\ & =\exp\left(j{\varphi_{W1D}}^{T}w\right) \end{array}$$

where $\varphi_{W1D}\in {\mathbb{C}}^{1\times M}$ denotes the fundamental phase error, and $w\in {\mathbb{R}}^{1\times N_{s}}$ denotes the weight. The vectors and matrix are given by:

(19) $$\begin{array}{cc} \varphi_{W1D}\left(m\right)=-\frac{4\pi f_{c}\Delta R\left(m\right)}{c}, & m=1,2,\ldots ,M \end{array}$$

(20) $$\begin{array}{cc} w\left(n_{s}\right)=\frac{f_{c}^{\prime }\left(n_{s}\right)}{f_{c}}, & n_{s}=1,2,\ldots ,N_{s} \end{array}$$

(21) $$\begin{array}{ll} D_{W1D}\left(m,n_{s}\right) & =\exp\left[j\varphi_{W1D}\left(m\right)w\left(n_{s}\right)\right]\\ & =\exp\left[-j\frac{4\pi \Delta R\left(m\right)f_{c}^{\prime }\left(n_{s}\right)}{c}\right] \end{array}.$$

The function exp(⋅) in Equation (18) is defined as follows:

(22) $$\exp\left(G\right)=\left[\begin{array}{cccc} e^{G_{1,1}} & e^{G_{1,2}} & \cdots & e^{G_{1,N}}\\ e^{G_{2,1}} & e^{G_{2,2}} & \cdots & e^{G_{2,N}}\\ \vdots & \vdots & & \vdots \\ e^{G_{M,1}} & e^{G_{M,2}} & \cdots & e^{G_{M,N}} \end{array}\right]$$

where $G=[G_{i,j}]\in {\mathbb{C}}^{M\times N}$ is an arbitrary matrix. In Equation (19), $\varphi_{W1D}\left(m\right)$ is proportional to ΔR(m), which is unknown and must be estimated in the phase error correction method.

For the narrow bandwidth system, the frequency interval Δf can be neglected, i.e., $n\cdot \Delta f<<f_{c}$. Therefore, Equation (20) can be approximated as

(23) $$\begin{array}{cc} w\left(n_{s}\right)\approx 1, & n_{s}=1,2,\ldots ,N_{s} \end{array}$$

Based on Equation (17), the received signal using random-frequency waveform with model error for the narrow bandwidth system will be expressed as:

(24) $$\begin{array}{ll} S_{\varepsilon } & =D_{W1D}⊙S\\ & =\exp\left(j{\varphi_{W1D}}^{T}w\right)⊙S\\ & \approx \exp\left\{{\left[j\varphi_{1D}\left(1\right),j\varphi_{1D}\left(2\right),\ldots ,j\varphi_{1D}\left(M\right)\right]}^{T}\left[\underset{N_{s}}{\underset{︸}{1,1,\ldots ,1}}\right]\right\}⊙S\\ & =\exp\left\{\mathrm{diag}\left[j\varphi_{1D}\left(1\right),j\varphi_{1D}\left(2\right),\ldots ,j\varphi_{1D}\left(M\right)\right]\right\}\cdot S\\ & =\mathrm{diag}\left[e^{j\varphi_{1D}\left(1\right)},e^{j\varphi_{1D}\left(2\right)},\cdots ,e^{j\varphi_{1D}\left(M\right)}\right]\cdot S\\ & =D_{1D}S \end{array}.$$

Comparing Equation (18) to Equation (24), the 1D phase error model is an approximation of the weighted 1D phase error model in the case of the narrow bandwidth system. Note that the former can only correct 1D phase error, while the latter can correct 2D phase error.

Similarly, the received signal using LFM waveform with model error for the narrow bandwidth system can also be written as:

(25) $$S_{\varepsilon }=D_{1D}S$$

where $S_{\varepsilon }\in {\mathbb{C}}^{M\times N}$ and $S\in {\mathbb{C}}^{M\times N}$ denote the echo data with and without phase error, respectively.

## 3. Phase Error Correction for Approximated Observation-Based CS-SAR Imaging

In this section, we will formulate the method to correct phase error for approximated observation-based CS-SAR imaging. First, some preliminary knowledge of approximated observation is reviewed. Then, a method is proposed to correct phase error for approximated observation-based CS-SAR imaging.

### 3.1. Approximated Observation

#### 3.1.1. Random-Frequency Waveform

Referring to [9,10], the approximated observation operators will be obtained by calculating the inverse of matched filtering (MF)-based algorithm. The Omega-K algorithm, which is a type of MF-based algorithm, can be used to achieve the stepped-frequency SAR imaging [11]. In this paper, the Omega-K algorithm is also used to achieve the imaging procedure of the random-frequency SAR, which can be expressed as:

(26) $$M\left(S\right)=F_{a}^{H}\left\{C\left[\left(F_{a}S\right)⊙H_{c}\Theta_{r}^{T}⊙H_{ref}\right]F_{r}^{H}\right\}$$

where $S\in {\mathbb{C}}^{M\times N_{s}}$ is the echo data without model error, C is the Stolt interpolation operator, $H_{c}$ is the range difference compensation function, and $H_{ref}$ is the reference function. The details of $H_{c}$ and $H_{ref}$ can be seen in [11]. F and $F^{H}$ are DFT matrix and inverse DFT matrix, respectively. The lower notation a and r are the azimuth and range direction, respectively. $\Theta_{r}$ can be found in Equation (4). The function $M:{\mathbb{C}}^{M\times N_{s}}\to {\mathbb{C}}^{M\times N}$ is the imaging procedure.

Based on the above exposition, the approximated observation operator I is obtained by calculating the inverse of imaging procedure as:

(27) $$I\left(G\right)=F_{a}^{H}\left\{C^{-1}\left[\left(F_{a}G\right)F_{r}\right]⊙H_{ref}^{*}\Theta_{r}⊙H_{c}^{*}\right\}$$

where $G\in {\mathbb{C}}^{M\times N}$ is the matrix form of g(x,y) in Equation (3), and $C^{-1}$ is the inverse of Stolt interpolation operator. The notation $I:{\mathbb{C}}^{M\times N}\to {\mathbb{C}}^{M\times N_{s}}$ denotes the approximated observation operator.

Based on Equation (27), we acquire the approximated observation-based CS-SAR model (named as CS-Omega-K [16,17]) as follow:

(28) $$\underset{G}{\min}\left\{{\left\|S-I\left(G\right)\right\|}_{F}^{2}+\lambda {\left\|G\right\|}_{1,1}\right\}$$

where ${\left\|\cdot \right\|}_{F}$ is the Frobenius norm of a matrix, and λ>0 is a regularization parameter. ${\left\|G\right\|}_{1,1}={\left\|\mathrm{vec}\left(G\right)\right\|}_{1}$ is the $l_{1,1}$ (pseudo) matrix norm [18]. The regularization item ${\left\|G\right\|}_{1,1}$ also can be substituted by ${\left\|\mathrm{vec}\left(G\right)\right\|}_{q}^{q}$ to enhance feature of imaging when 0≤q≤1. In this paper, we simply set q=1, since the widely used soft-thresholding corresponds to q=1 and the soft-thresholding operator can be analytically specified.

#### 3.1.2. LFM Waveform

In [10,19], the chirp scaling algorithm, which is also a type of MF-based algorithm, was used to achieve the LFM SAR imaging. In this paper, the chip scaling algorithm is used to achieve the imaging procedure of the LFM SAR, which can be expressed as:

(29) $$M\left(S\right)=F_{a}^{H}\left\{H_{3}⊙\left\{H_{2}⊙\left[H_{1}⊙\left(F_{a}S\right)F_{r}\right]F_{r}^{H}\right\}\right\}$$

where $S\in {\mathbb{C}}^{M\times N}$ denotes the echo data without model error. $H_{1}$, $H_{2}$ and $H_{3}$ are the standard phase functions of the chirp scaling [19] ($\Phi_{1}$, $\Phi_{2}$ and $\Phi_{3}$). The function $M:{\mathbb{C}}^{M\times N}\to {\mathbb{C}}^{M\times N}$ is the imaging procedure.

Based on the above exposition, the approximated observation I is obtained by calculating the inverse of imaging procedure as:

(30) $$I\left(G\right)=F_{a}^{H}\left\{H_{1}^{*}⊙\left\{H_{2}^{*}⊙\left[H_{3}^{*}⊙\left(F_{a}G\right)F_{r}\right]F_{r}^{H}\right\}\right\}$$

where $G\in {\mathbb{C}}^{M\times N}$ is the matrix form of g(x,y) in Equation (10). The notation $I:{\mathbb{C}}^{M\times N}\to {\mathbb{C}}^{M\times N}$ denotes the approximated observation operator.

Based on Equation (30), we are able to acquire the approximated observation-based CS-SAR model (named as CS-chirp scaling [10]) as follow:

(31) $$\underset{G}{\min}\left\{{\left\|S-I\left(G\right)\right\|}_{F}^{2}+\lambda {\left\|G\right\|}_{1,1}\right\}.$$

The aim of briefing the published research is to suggest a new phase error correction method, which will be formulated as follows and can achieve the phase error correction for approximated observation-based CS-SAR imaging.

### 3.2. 1D Phase Error Correction for Approximated Observation-Based CS-SAR Imaging

Thus far, we have derived the inexact (nominal) observation model, but the model error is still unknown. Thus, CS-SAR imaging models must be modified. Considering the 1D phase error models in Equations (24) and (25), the CS-SAR imaging models in Equation (28) using random-frequency waveform and in Equation (31) using LFM waveform will be modified as:

(32) $$\underset{G,D_{1D}}{\min}\left\{{\left\|S_{\varepsilon }-D_{1D}I\left(G\right)\right\|}_{F}^{2}+\lambda {\left\|G\right\|}_{1,1}\right\}.$$

The cost function is denoted by:

(33) $$J\left(G,D_{1D}\right)={\left\|S_{\varepsilon }-D_{1D}I\left(G\right)\right\|}_{F}^{2}+\lambda {\left\|G\right\|}_{1,1}$$

where the phase error $D_{1D}$ is written, referring to Equation (24), as:

(34) $$D_{1D}=\mathrm{diag}\left[e^{j\varphi_{1D}\left(1\right)},e^{j\varphi_{1D}\left(2\right)},\cdots ,e^{j\varphi_{1D}\left(M\right)}\right].$$

By solving Equation (32), both the image and the 1D phase error can be jointly obtained. Using similar methods proposed in the [5,8], both the image and phase error are estimated by solving a two-step optimization problem. In the first step, G is estimated by minimizing the cost function Equation (33) using the given $D_{1D}$. In the second step, $D_{1D}$ is obtained by minimizing the cost function using the estimated G. By iteratively updating G and $D_{1D}$ as described above, both the image and the 1D phase error can be jointly estimated. The algorithm flow is outlined as Algorithm 1. Initially, let

(35) $$D_{1D}=\mathrm{diag}\left[\underset{M}{\underset{︸}{1,1,\cdots ,1}}\right],$$

i.e., $\begin{array}{cc} \varphi_{1D}\left(m\right)=0, & m=1,2,\ldots ,M \end{array}$.

**Algorithm 1.** 1D Phase Error Correction for Approximated Observation-Based CS-SAR Imaging.Initialize: i=0, $D_{1D}^{0}=\mathrm{diag}\left[1,1,\ldots ,1\right]$
Step 1: Image reconstruction $G^{i+1}={\mathrm{argmin}}_{G}J\left(G,D_{1D}^{i}\right)$
Step 2: Phase error estimation $D_{1D}^{i+1}={\mathrm{argmin}}_{D_{1D}}J\left(G^{i+1},D_{1D}\right)$
Step 3: Let i=i+1 and return to step 1.   Terminate when i is equal to a preset threshold $I_{\max}$.

Steps 1 and 2 are the major steps of Algorithm 1. In Step 1, the image is reconstructed by the given phase error. The optimization problem is expressed as:

(36) $$\begin{array}{ll} G^{i+1} & ={\mathrm{argmin}}_{G}J\left(G,D_{1D}^{i}\right)\\ & ={\mathrm{argmin}}_{G}\left\{{\left\|S_{\varepsilon }-D_{1D}^{i}I\left(G\right)\right\|}_{F}^{2}+\lambda {\left\|G\right\|}_{1,1}\right\} \end{array}$$

Since $D_{1D}^{i}$ is a diagonal matrix, seen in Equation (34), Equation (36) can be rewritten as:

(37) $$G^{i+1}={\mathrm{argmin}}_{G}\left\{{\left\|{D_{1D}^{i}}^{*}S_{\varepsilon }-I\left(G\right)\right\|}_{F}^{2}+\lambda {\left\|G\right\|}_{1,1}\right\}$$

Then, due to the linearity of I, we solve the optimization problem efficiently by iterative thresholding algorithm (ITA) [9,10,20]. Let $G^{0}=0$, and the optimal solution is obtained by using the iteration:

(38) $$G^{i+1}=E_{1,\lambda \mu }\left(G^{i}+\mu M\left({D_{1D}^{i}}^{*}S_{\varepsilon }-I\left(G^{i}\right)\right)\right)$$

where μ is step size, which controls the convergence of the ITA. In Equation (38), $E_{1,\lambda \mu }$ is a thresholding operator, which is defined as:

(39) $$E_{1,\lambda \mu }\left(G\right)=\left[\begin{array}{cccc} E_{1,\lambda \mu }\left(G_{1,1}\right) & E_{1,\lambda \mu }\left(G_{1,2}\right) & \cdots & E_{1,\lambda \mu }\left(G_{1,N}\right)\\ E_{1,\lambda \mu }\left(G_{2,1}\right) & E_{1,\lambda \mu }\left(G_{2,2}\right) & \cdots & E_{1,\lambda \mu }\left(G_{2,N}\right)\\ \vdots & \vdots & & \vdots \\ E_{1,\lambda \mu }\left(G_{M,1}\right) & E_{1,\lambda \mu }\left(G_{M,2}\right) & \cdots & E_{1,\lambda \mu }\left(G_{M,N}\right) \end{array}\right]$$

where soft-thresholding operator $E_{1,\lambda \mu }$ is analytically specified as:

(40) $$E_{1,\lambda \mu }\left(x\right)=\{\begin{array}{ll} \mathrm{sign}\left(x\right)\left(\left|x\right|-\lambda \mu \right), & \mathrm{if}\;\left|x\right|\geq \lambda \mu ,\\ 0, & \mathrm{otherwise}. \end{array}$$

In the proposed method, we simply set μ=1 ($0<\mu <{\left\|I\right\|}_{2}^{2}$ [9]). With fixed parameter μ, the parameter λ will depend on the sparsity parameter $K_{0}$. We set $\lambda ={\left|G\right|}_{K_{0}+1}/\mu$ (${\left|G\right|}_{K_{0}}$ is the $K_{0}$-th largest component of G in magnitude).

In Step 2, the phase error is estimated by the reconstructed image. The optimization problem is expressed as:

(41) $$\begin{array}{ll} D_{1D}^{i+1} & ={\mathrm{argmin}}_{D_{1D}}J\left(G^{i+1},D_{1D}\right)\\ & ={\mathrm{argmin}}_{D_{1D}}\left\{{\left\|S_{\varepsilon }-D_{1D}I\left(G^{i+1}\right)\right\|}_{F}^{2}+\lambda {\left\|G^{i+1}\right\|}_{1,1}\right\} \end{array}.$$

Since $\lambda {\left\|G^{i+1}\right\|}_{1,1}$ is a constant, Equation (41) is rewritten as:

(42) $$D_{1D}^{i+1}={\mathrm{argmin}}_{D_{1D}}{\left\|S_{\varepsilon }-D_{1D}I\left(G^{i+1}\right)\right\|}_{F}^{2}.$$

From Equations (34) and (42), it is quite obvious that the data error of the m-th observation position is only related to $\varphi_{1D}\left(m\right)$. Then, the optimization problem Equation (42) is divided into a set of independent optimization problems as follows:

(43) $$\varphi_{1D}^{i+1}\left(m\right)={\mathrm{argmin}}_{\varphi_{1D}\left(m\right)}{\left\|{\left[S_{\varepsilon }\right]}_{m}-e^{j\varphi_{1D}\left(m\right)}{\left[I\left(G^{i+1}\right)\right]}_{m}\right\|}_{2}^{2}$$

where notation ${\left[\cdot \right]}_{m}$ denotes the m-th row of the matrix. The phase error $\varphi_{1D}^{i+1}\left(m\right)$ and/or $D_{1D}^{i+1}\left(m,m\right)$ can be obtained as follows (the details can be found in the Appendix A):

(44) $$\varphi_{1D}^{i+1}\left(m\right)=\mathrm{angle}\left({\left[S_{\varepsilon }\right]}_{m}\cdot {\left[I\left(G^{i+1}\right)\right]}_{m}^{H}\right)$$

Substituting Equation (44) into Equation (34), Step 2 of Algorithm 1 can be implemented straightforwardly.

### 3.3. Weighted 1D Phase Error Correction for Approximated Observation-Based CS-SAR Imaging

Considering the SAR imaging system using random-frequency waveform without narrow bandwidth approximation, the SAR imaging model (28) is modified as:

(45) $$\underset{G,D_{W1D}}{\min}\left\{{\left\|S_{\varepsilon }-D_{W1D}⊙I\left(G\right)\right\|}_{F}^{2}+\lambda {\left\|G\right\|}_{1,1}\right\}.$$

The cost function is denoted by

(46) $$J\left(G,D_{W1D}\right)={\left\|S_{\varepsilon }-D_{W1D}⊙I\left(G\right)\right\|}_{F}^{2}+\lambda {\left\|G\right\|}_{1,1}$$

where $D_{W1D}\in {\mathbb{C}}^{M\times N_{s}}$ can be seen in Equation (18). By solving Equation (45), both the image and the weighted 1D phase error will be estimated jointly. The method is similar to Algorithm 1, and the algorithm flow is outlined in Algorithm 2. In the initialization, let

(47) $$D_{W1D}=\mathrm{ones}\left(M,N_{s}\right)=\left[\begin{array}{cccc} 1 & 1 & \cdots & 1\\ 1 & 1 & \cdots & 1\\ \vdots & \vdots & & \vdots \\ 1 & 1 & \cdots & 1 \end{array}\right],$$

i.e., $\begin{array}{cc} \varphi_{W1D}\left(m\right)=0, & m=1,2,\cdots ,M \end{array}$.

**Algorithm 2.** Weighted 1D Phase Error Correction for Approximated Observation-Based CS-SAR Imaging.Initialize: i=0, $D_{W1D}^{0}=\mathrm{ones}\left(M,N_{s}\right)$
Step 1: Image reconstruction $G^{i+1}={\mathrm{argmin}}_{G}J\left(G,D_{W1D}^{i}\right)$
Step 2: Phase error estimation $D_{W1D}^{i+1}={\mathrm{argmin}}_{D_{W1D}}J\left(G^{i+1},D_{W1D}\right)$
Step 3: Let i=i+1 and return to step 1.   Terminate when i is equal to a preset threshold $I_{\max}$.

In Step 1, the optimization problem is expressed as:

(48) $$\begin{array}{ll} G^{i+1} & ={\mathrm{argmin}}_{G}J\left(G,D_{W1D}^{i}\right)\\ & ={\mathrm{argmin}}_{G}\left\{{\left\|S_{\varepsilon }-D_{W1D}^{i}⊙I\left(G\right)\right\|}_{F}^{2}+\lambda {\left\|G\right\|}_{1,1}\right\}\\ & ={\mathrm{argmin}}_{G}\left\{{\left\|{D_{W1D}^{i}}^{*}⊙S_{\varepsilon }-I\left(G\right)\right\|}_{F}^{2}+\lambda {\left\|G\right\|}_{1,1}\right\} \end{array}.$$

Let $G^{0}=0$, and use the iteration

(49) $$G^{i+1}=E_{1,\lambda \mu }\left(G^{i}+\mu M\left({D_{W1D}^{i}}^{*}⊙S_{\varepsilon }-I\left(G^{i}\right)\right)\right)$$

Then, the optimization problem (48) is efficiently solved by the same method as Algorithm 1.

In Step 2, the optimization problem is expressed as:

(50) $$\begin{array}{ll} D_{W1D}^{i+1} & ={\mathrm{argmin}}_{D_{W1D}}J\left(G^{i+1},D_{W1D}\right)\\ & ={\mathrm{argmin}}_{D_{W1D}}\left\{{\left\|S_{\varepsilon }-D_{W1D}⊙I\left(G^{i+1}\right)\right\|}_{F}^{2}+\lambda {\left\|G^{i+1}\right\|}_{1,1}\right\}\\ & ={\mathrm{argmin}}_{D_{W1D}}{\left\|S_{\varepsilon }-D_{W1D}⊙I\left(G^{i+1}\right)\right\|}_{F}^{2} \end{array}.$$

From Equations (18) and (50), it can be seen that the data error of the m-th observation position is only related to $\varphi_{W1D}\left(m\right)$. Then, the optimization problem in Equation (50) can also be divided into a set of independent optimization problems as follows:

(51) $$\varphi_{W1D}^{i+1}\left(m\right)={\mathrm{argmin}}_{\varphi_{W1D}\left(m\right)}{\left\|{\left[S_{\varepsilon }\right]}_{m}-\exp\left(j\varphi_{W1D}\left(m\right)\cdot w\right)⊙{\left[I\left(G^{i+1}\right)\right]}_{m}\right\|}_{2}^{2}.$$

The weight w, which is a priori knowledge, must be obtained before solving Equation (51). Equation (51) is an unconstrained optimization problem, which can be solved using gradient-based optimization method [8]. In Equation (51), there is only one unknown $\varphi_{W1D}\left(m\right)$ to be estimated and it belongs to a real number. Hence, the optimal solution is able to be obtained by line search [21].

### 3.4. Memory Cost

The proposed method consists of a two-step optimization problem. In the first step, the image is reconstructed. In the second step, the phase error is estimated and compensated. The second step is divided into a set of independent optimization problems. Thus the biggest matrix will appear in the first step. There are some notations to be denoted as follows. M, N and K denote the number of azimuth samples, the number of range samples, and the number of scatterer points after discretizing the scene, respectively. The size of the sensing matrix in the phase error correction for exact observation based CS-SAR imaging is MN×K complex numbers, but the size of the matrix in the proposed method is M×N complex numbers. Therefore, the proposed method can reduce the memory cost and the hardware requirement significantly.

### 3.5. Convergence

The proposed method is achieved by calculating a two-step optimization problem. The cost function of 1D phase error correction or weighted 1D phase error correction for approximated observation-based CS-SAR imaging can be denoted as J(G,D).

In the first step, $G^{i+1}={\mathrm{argmin}}_{G}J\left(G,D^{i}\right)$, and thus $J\left(G^{i+1},D^{i}\right)\leq J\left(G^{i},D^{i}\right)$.

In the second step, $D^{i+1}={\mathrm{argmin}}_{D}J\left(G^{i+1},D\right)$, i.e., $J\left(G^{i+1},D^{i+1}\right)\leq J\left(G^{i+1},D^{i}\right)$.

Combining the two steps, there will be $J\left(G^{i+1},D^{i+1}\right)\leq J\left(G^{i},D^{i}\right)$, and then the cost function J(G,D) is a monotonous decrease function. Since J(G,D)≥0, the convergence of this process can be guaranteed.

### 3.6. Computational Complexity

In order to calculate the computational complexity, we have defined some notations, which are the number of azimuth samples M, the number of range samples N, the number of required iteration in the first step $I_{1}$, and the number of required iteration in algorithm $I_{\max}$.

In the first step, the image is reconstructed. This process is an iterative process. One-step iteration includes the calculations of imaging procedure, approximated observation procedure, and thresholding operation. Both imaging procedure and approximated observation procedure have the same computational complexity of $O\left[MN\log_{2}\left(MN\right)\right]$. The complexity of thresholding operator is order O(MN). Thus, the complexity of the first step is at the order $O\left[I_{1}MN\log_{2}\left(MN\right)\right]$.

In the second step, the phase error is estimated and compensated. The optimal solution of the second step in Algorithm 1 can be analytically expressed. The complexity of this procedure is order O(MN). The optimal solution of the second step in Algorithm 2 is able to be obtained by line search. The complexity of this procedure is order $O\left(I_{2}MN\right)$, where $I_{2}$ denotes the number of iteration in line search.

Based on the above exposition, the computational complexities of Algorithms 1 and 2 are at the order $O\left[I_{\max}I_{1}MN\log_{2}\left(MN\right)\right]$ and $O\left\{I_{\max}MN\left[I_{1}\log_{2}\left(MN\right)+I_{2}\right]\right\}$, respectively.

## 4. Simulation and Experimental Results

In this section, we will present a series of simulation and experimental results to verify the effectiveness of the proposed method. All experiments are performed using MATLAB R2015a on a PC equipped with an Intel Core i5-4590 CPU (3.30 GHz and 8 GB memory, University of Science and Technology of China, Hefei, China).

### 4.1. 1D Phase Error Correction for Approximated Observation-Based CS-SAR Imaging

#### 4.1.1. Simulation Results

The simulation parameters for the random-frequency waveform are shown in Table 1. We then choose a sparse imaging scene consisting of four-point scatterers, the positions of which are shown in Table 2. In order to make each observation position have the same data volume to estimate phase error, the random-frequency points are selected as follows: in one observation position, the frequency points are selected randomly from stepped-frequency points, and then this selecting scheme is utilized in each observation position. The raw data are first generated in the time domain by exact observation. Then, we add the Gaussian noise and the 1D phase error to the data. The signal-to-noise ratio (SNR) is 20 dB. Two types of phase error are 1D quadratic phase error and 1D random phase error. The 1D quadratic phase error is distributed in [−π/2, +π/2], and the 1D random phase error is uniformly distributed in [−0.8π, +0.8π].

**Figure 2:**
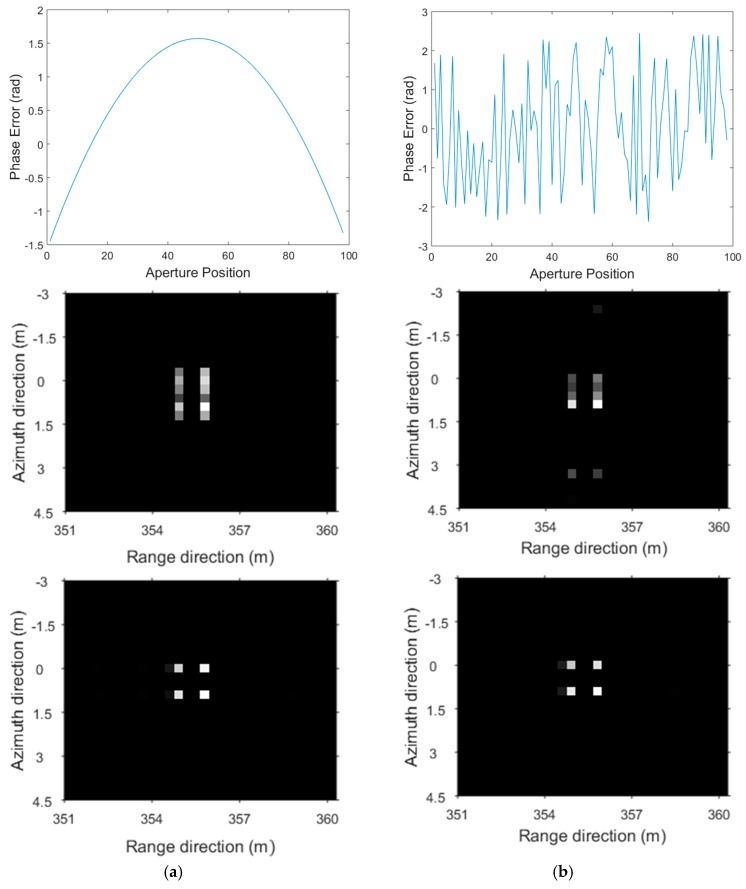
Results for the random-frequency waveform. (**Top**) 1D quadratic phase error and 1D random phase error. (**Middle**) Results of CS-Omega-K without phase error correction. (**Bottom**) Results of Algorithm 1. (**a**) Results for 1D quadratic phase error; and (**b**) results for 1D random phase error.

Figure 2 shows the phase error distribution and the imaging results for the random-frequency waveform. Figure 2a shows the results of the 1D quadratic phase error, and Figure 2b shows the results of the 1D random phase error. The top subfigures show the 1D phase error added to the raw data. The middle subfigures show the results of approximated observation-based CS-SAR imaging (named as CS-Omega-K [16,17]) without phase error correction. The bottom subfigures show the results of Algorithm 1. For both imaging methods, the approximated observation operator is acquired from the inverse of Omega-K algorithm, and the sparsity is set to $K_{0}=12$, which determines the threshold of ITA.

In the middle subfigures, the images of point targets are defocused in the azimuth direction due to the 1D phase error. In the bottom subfigures, the results show the effectiveness of Algorithm 1, which can correct the 1D phase error and focus the images better in the azimuth direction.

Then, we conduct experiments to compare the performance of the proposed method with the existing method [8]. The SNR is 20 dB, and the extent of 1D random phase error is 0.1π.

**Figure 3:**
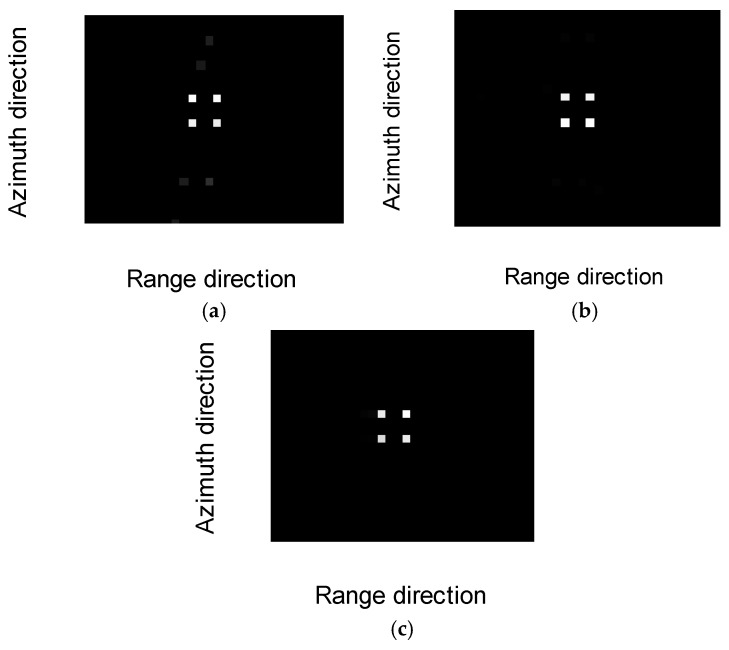
Imaging results of the different methods: (**a**) reconstruction result without phase error correction; (**b**) reconstruction result with compensation of observation position errors; and (**c**) reconstruction result of the proposed method (Algorithm 1).

Figure 3 shows the imaging results of the proposed method and other existing methods. Figure 3a shows the reconstruction result without phase error correction. Figure 3b shows the reconstruction result with compensation of observation position errors [8]. Figure 3c shows the result of the proposed method (Algorithm 1). In Figure 3a, it can be seen that the 1D phase error will defocus the reconstructed image in the azimuth direction without phase error correction. Both the proposed method and the existing method can correct phase error and focus image better in Figure 3b,c, but the proposed method requires much less memory cost referring to Section 3.4. In the simulation of the proposed method, the numbers of azimuth samples and range samples are M=98 and N=1536, respectively. Thus, the size of the matrix in the proposed method is M×N=98×1536 complex numbers, and the memory requirement is 1.15 MB. In the simulation of the existing method, the scene needs to be discretized, which is taken as 40×40. Thus, the number of scatterer points after discretizing the scene is K=1600. Therefore, the size of the matrix in the existing method is MN×K=98×1536×1600 complex numbers, and the memory requirement is 1.79 GB.

Next, we perform a series of one-point simulations for investigating the influence of the sparsity parameter $K_{0}$ and the noise size to the proposed algorithm. The imaging scene is composed of one point scatterer. Then, the target-to-background ratio (TBR) [5] can be used as a criterion to evaluate the performance of the proposed method conveniently. The TBR of a reconstructed image can be given as follows:

(52) $$\mathrm{TBR}\left(X\right)=20\log_{10}\left(\frac{\max_{i\in T}\left|X_{i}\right|}{\frac{1}{I_{B}}\sum_{j\in B}\left|X_{j}\right|}\right)$$

where T and B denote the pixel indices for the target and the background regions, respectively. $I_{B}$ is the number of background pixels.

The extent of 1D random phase error is varied from [−0.2π,+0.2π] to [−0.5π,+0.5π]. First, the sparsity parameter $K_{0}$ is changed from 3 to 11. The SNR is 20 dB. Second, we simulate the cases of different SNR from 0 dB to 30 dB. The sparsity parameter $K_{0}$ is set to 4. Other simulation parameters are the same as Table 1.

**Figure 4:**
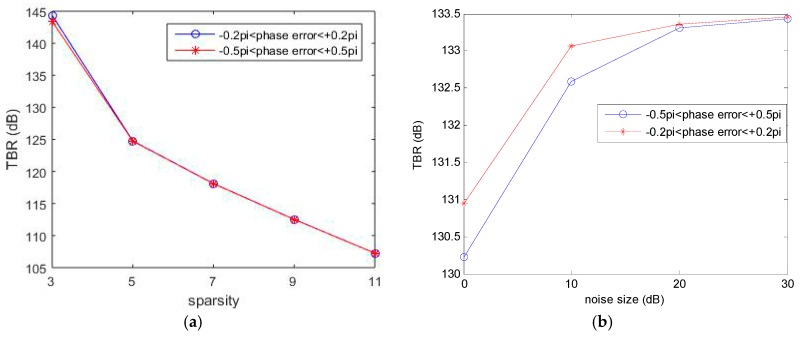
Imaging performance with respect to: the sparsity parameter (**a**); and the noise size (**b**).

Figure 4 shows the behavior of TBR values with respect to the sparsity parameter and the noise size, respectively. In Figure 4a, the TBR decreases with the increase of sparsity parameter. Therefore, exact prior knowledge can contribute to the choice of sparsity parameter positively. In Figure 4b, the TBR increases as the SNR increases. Note that there is an insignificant difference between different phase error sizes in the case of high SNR.

#### 4.1.2. Experimental Results

The RADARSAT-1 is a well-known satellite SAR using LFM waveform, and the raw data were collected on 16 June 2002 about Vancouver region. The experimental parameters for the LFM waveform are shown in Table 3 [9,12].

In the region of English Bay, there are four sparsely distributed vessels, thus the region is a typically sparse scene. Then, the proposed method (Algorithm 1) can be applied to reconstruction of the scene. First, the chirp scaling algorithm, which is one type of MF-based algorithm, is applied to reconstruction of the region of English Bay. Second, approximated observation-based CS-SAR imaging method (named as CS-chirp scaling [10]) without phase error correction is applied, where the approximated observation operator is acquired from the inverse of chirp scaling algorithm. We set the sparsity to $K_{0}=10,000$. Third, Algorithm 1 is utilized to reconstruct the scene, where the same approximated observation operator and the same sparsity are used.

**Figure 5:**
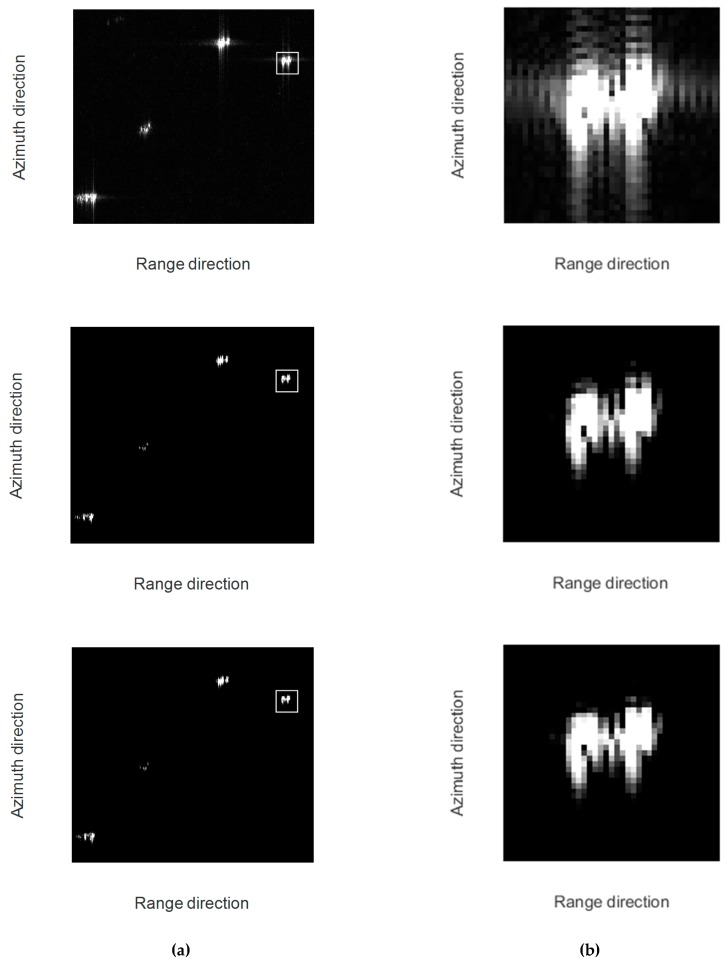
Results for the LFM waveform. (**Top**) Results of MF-based algorithm (chirp scaling). (**Middle**) Results of CS-chirp scaling without phase error correction. (**Bottom**) Results of Algorithm 1. (**a**) Application results on RADARSAT-1 (region of English Bay). (**b**) Detailed comparison on the selected area.

Figure 5a,b shows the results of the three methods and the enlarged region, respectively. In the top subfigures of Figure 5, the chirp scaling algorithm reconstructs the scene with serious sidelobes. In the middle subfigures, it can be seen that CS-chirp scaling can suppress sidelobes and improve resolution. In the bottom subfigures, the reconstructed scene can be focused better in the azimuth direction. Thus, Algorithm 1 can not only suppress sidelobes, but also achieve phase error compensation.

We further use the image entropy [22] as a criterion to quantitatively evaluate the performance of the three methods. The entropy of an image is defined as follows:

(53) $$\mathrm{Entropy}\left(X\right)=-\sum_{i}p_{i}\log_{2}\left(p_{i}\right)$$

where $p_{i}$ is the histogram of the recovered gray level image.

**Figure 6:**
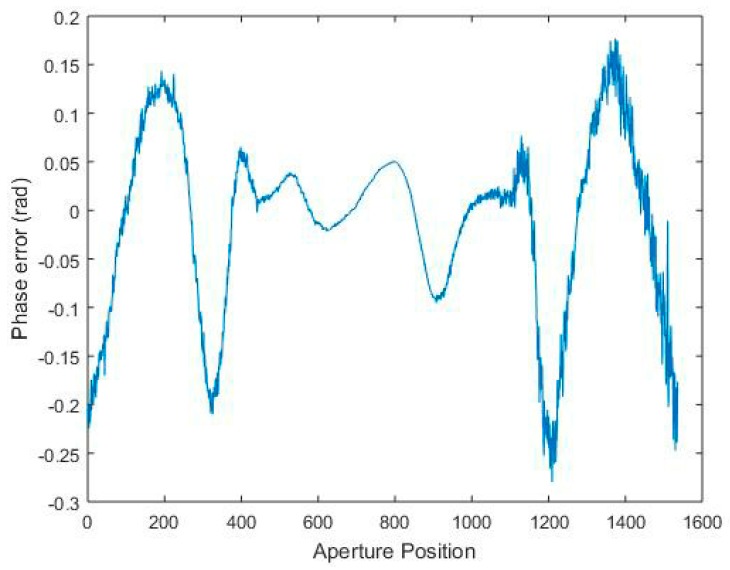
1D phase error estimation obtained by the proposed method in each aperture position.

The entropy values of the reconstructed images by the three methods are shown in Table 4. It can be seen that the proposed method (Algorithm 1) has a lower entropy, and thereby exhibits a better performance of image reconstruction. In Table 4, there is no significant difference in entropy between CS-chirp scaling method and the proposed method (Algorithm 1), since the estimated phase error (shown in Figure 6) is not too large. The trajectory of the satellite is much more stable than that of an airplane, and thus the phase error is smaller. If the phase error arising from observation position uncertainties is large enough, there will be a significant difference.

### 4.2. Weighted 1D Phase Error Correction for Approximated Observation-Based CS-SAR Imaging

Furthermore, with narrow bandwidth approximation, the phase error of echo data along range time is constant for one observation position, which can be seen in Equation (34). However, considering the SAR imaging system using random-frequency waveform without narrow bandwidth approximation, the phase error of echo data along range time is different for one observation position, which can be seen in Equation (18). The number of unknowns for weighted 1D phase error model is also *M*, which is the same as the 1D phase error model. The simulation parameters are the same as Table 1. We then set one point scatterer. The position of the target is shown in Table 5. The raw data are first generated in the time domain by exact observation. Then, we add the Gaussian noise and the weighted 1D phase error to the data. The SNR is 20 dB. Two types of phase error $\varphi_{W1D}\in {\mathbb{C}}^{M\times 1}$ are utilized in simulations. The extent of the error is 1/8 of the wavelength so that the weighted 1D quadratic phase error is distributed in [−π/8,+π/8], and the weighted 1D random phase error is uniformly distributed in [−π/8,+π/8]. The weight $w\in {\mathbb{C}}^{1\times N_{s}}$ depends on the selected frequency points.

The imaging scene is composed of one point scatterer. Then, the TBR can also be used as a criterion to evaluate the performance of the two methods conveniently.

The images are reconstructed by the two imaging methods with sparsity $K_{0}=5$. TBR values of the reconstructed images by the Algorithm 1 and Algorithm 2 are listed in Table 6. It can be seen that the Algorithm 2 has a higher TBR than Algorithm 1, and thereby Algorithm 2 has a better performance.

For presenting the effect of the weight $w\in {\mathbb{C}}^{1\times N_{s}}$, we change the frequency interval from 0.33 MHz to 0.66 MHz, and then the bandwidth will be changed to 1024 MHz. Other simulation parameters are the same as Table 1. TBR values with larger frequency interval are given in Table 7. Comparing Table 6 with Table 7, it can be observed that there will be more significant difference under the case of the larger frequency interval. Accordingly, the weighted 1D phase error correction for CS-SAR imaging will be more effective in the case of large frequency interval.

Further, we increase the extent of phase error and then conduct the simulations. The weighted 1D quadratic phase error is distributed in [−π/2,+π/2], and the weighted 1D random phase error is uniformly distributed in [−π/2,+π/2]. The simulation parameters are the same as Table 1. TBR values with a larger extent of phase error are shown in Table 8. Comparing Table 6 with Table 8, it can be observed that there is a more significant difference under the case with a larger extent of phase error.

## 5. Conclusions

In this paper, we proposed a phase error correction method for approximated observation-based CS-SAR imaging. The proposed method yields a clear advantage in term of memory cost over conventional CS-based autofocus algorithms. The 1D phase error model, which can be conveniently utilized in autofocus technique without any a priori knowledge, is based on narrow bandwidth approximation. We also analyzed the inherent relationship between the geometric model and the phase error model in the case of random-frequency waveform. By incorporating a priori knowledge, the weighted 1D phase error model was proposed, which corrects the 2D phase error by estimating a 1D problem. The proposed weighted 1D phase error model is more precise than the 1D phase error model, thus has a better performance.

Although the weighted 1D phase error correction method proposed in this work can be applied only in the case of random-frequency waveform rather than the LFM one, since different waveforms have different geometry and signal models, it would be possible to apply the weighted 1D phase error correction to LFM waveform with appropriate modification, and this will be our future effort.

## Figures and Tables

**Table 1:** Simulation parameters for the random-frequency waveform.

Paramater	Value
Center Frequency	5 GHz
Bandwidth	512 MHz
Frequency Interval	0.33 MHz
Frequency Number	1536
Pulse Time Interval	4 × 10^−6^ s
Radar Velocity	50 m/s
Azimuth Beam Width	4.3°
Squint Angle	0°
Scene Center Range	400 m
Number of Sequences	98
Number of Selected Frequencies	154

**Table 2:** Target positions.

	Azimuth (m)	Range (m)
Target 1	0	354.9
Target 2	0.9	354.9
Target 3	0	355.8
Target 4	0.9	355.8
Scene Center	0	400

**Table 3:** Experimental parameters for the RADARSAT-1.

Paramater	Value
Sampling Rate	32.317 MHz
Range FM Rate	0.72135 MHz/μs
Pulse Duration	41.74 μs
Radar Center Frequency	5.300 GHz
Pulse Repetition Frequency	1256.98 Hz
Effective Radar Velocity	7062 m/s
Azimuth FM Rate	1733 Hz/s

**Table 4:** Entropy values by different methods.

	Chirp Scaling	CS-Chirp Scaling	Algorithm 1
Entropy	1.94780	8.792 × 10^−2^	8.717 × 10^−2^

**Table 5:** Target positions.

	Azimuth (m)	Range (m)
Target	0	355.8

**Table 6:** TBR values by different methods.

	Algorithm 1	Algorithm 2	Difference between Two Methods
Quadratic phase error	1.2936 × 10^2^	1.2947 × 10^2^	0.11
Random phase error	1.3149 × 10^2^	1.3161 × 10^2^	0.12

**Table 7:** TBR values by different methods with larger frequency interval.

	Algorithm 1	Algorithm 2	Difference between Two Methods
Quadratic phase error	1.1562 × 10^2^	1.1592 × 10^2^	0.30
Random phase error	1.1596 × 10^2^	1.1628 × 10^2^	0.32

**Table 8:** TBR values by different methods with a larger extent of phase error.

	Algorithm 1	Algorithm 2	Difference between Two Methods
Quadratic phase error	1.0736 × 10^2^	1.2967 × 10^2^	22.31
Random phase error	1.2698 × 10^2^	1.3128 × 10^2^	4.30

## Appendix A

In this appendix, we will derive Equation (44) from Equation (43). The optimization problem, Equation (43), is as follows:

$$\varphi_{1D}^{i+1}\left(m\right)={\mathrm{argmin}}_{\varphi_{1D}\left(m\right)}{\left\|{\left[S_{\varepsilon }\right]}_{m}-e^{j\varphi_{1D}\left(m\right)}{\left[I\left(G^{i+1}\right)\right]}_{m}\right\|}_{2}^{2}$$

For simplifying the notations, let $a={\left[S_{\varepsilon }\right]}_{m}\in {\mathbb{C}}^{1\times N}$, $\theta =\varphi_{1D}\left(m\right)$ and $b={\left[I\left(G^{i+1}\right)\right]}_{m}\in {\mathbb{C}}^{1\times N}$. Then, the cost function can be simplified as:

‖[Sε]m−ejφ1D(m)⋅[I(Gi+1)]m‖22=‖a−ejθb‖22=(a−ejθb)(a−ejθb)H=aaH+bbH−ejθbaH−a(ejθb)H=aaH+bbH−2Re[a(ejθb)H]=aaH+bbH−2Re[e−jθabH]

Since argmaxθRe[e−jθabH]=angle(abH), the equation becomes

$$\arg\min_{\theta }{\left\|a-e^{j\theta }b\right\|}_{2}^{2}=\mathrm{angle}\left(ab^{H}\right)$$

Finally, we can get

$$\begin{array}{ll} \varphi_{1D}^{i+1}\left(m\right) & ={\mathrm{argmin}}_{\varphi_{1D}\left(m\right)}{\left\|{\left[S_{\varepsilon }\right]}_{m}-e^{j\varphi_{1D}\left(m\right)}{\left[I\left(G^{i+1}\right)\right]}_{m}\right\|}_{2}^{2}\\ & =\mathrm{angle}\left(ab^{H}\right)\\ & =\mathrm{angle}\left({\left[S_{\varepsilon }\right]}_{m}\cdot {\left[I\left(G^{i+1}\right)\right]}_{m}^{H}\right) \end{array}$$
